# Genetic regulation and manipulation of nicotine biosynthesis in tobacco: strategies to eliminate addictive alkaloids

**DOI:** 10.1093/jxb/erad341

**Published:** 2023-08-30

**Authors:** Tsubasa Shoji, Takashi Hashimoto, Kazuki Saito

**Affiliations:** Instutute of Natural Medicine, University of Toyama, Sugitani, Toyama, Toyama 930-0194, Japan; RIKEN Center for Sustainable Resource Science, Tsurumi-ku, Yokohama, Kanagawa 230-0045, Japan; Nara Institute of Science and Technology, Ikoma, Nara 630-0192, Japan; RIKEN Center for Sustainable Resource Science, Tsurumi-ku, Yokohama, Kanagawa 230-0045, Japan; French Alternative and Atomic Energy Commission, France

**Keywords:** Alkaloid, biosynthesis, jasmonate, *Nicotiana*, nicotine, tobacco, transcription factor

## Abstract

Tobacco (*Nicotiana tabacum* L.) is a widely cultivated crop of the genus *Nicotiana*. Due to the highly addictive nature of tobacco products, tobacco smoking remains the leading cause of preventable death and disease. There is therefore a critical need to develop tobacco varieties with reduced or non-addictive nicotine levels. Nicotine and related pyridine alkaloids biosynthesized in the roots of tobacco plants are transported to the leaves, where they are stored in vacuoles as a defense against predators. Jasmonate, a defense-related plant hormone, plays a crucial signaling role in activating transcriptional regulators that coordinate the expression of downstream metabolic and transport genes involved in nicotine production. In recent years, substantial progress has been made in molecular and genomics research, revealing many metabolic and regulatory genes involved in nicotine biosynthesis. These advances have enabled us to develop tobacco plants with low or ultra-low nicotine levels through various methodologies, such as mutational breeding, genetic engineering, and genome editing. We review the recent progress on genetic manipulation of nicotine production in tobacco, which serves as an excellent example of plant metabolic engineering with profound social implications.

## Teaser

Molecular and genomics studies have revealed metabolic and regulatory genes involved in nicotine biosynthesis, enabling us to develop tobacco plants with ultra-low nicotine levels through mutational breeding, genetic engineering, and genome editing.

## Introduction

Tobacco (*Nicotiana tabacum* L.) is an economically important crop that is cultivated around the world, with a global production of ~7.1 Mt in 2020; the top producing countries include China, India, Brazil, and the USA (https://ourworldindata.org/grapher/tobacco-production?time=latest). The genus *Nicotiana*, which is part of the family *Solanaceae* and comprises 76 naturally occurring species (Knapp *et al.*, 2004), is one of the most extensively studied genera of flowering plants, in large part due to its economic and cultural importance (Lewis, 2011). *Nicotiana* species arose in the Americas and Australia, where they have been traditionally used by native peoples for recreational and therapeutic purposes (Goodman, 2004).

After Columbus arrived in the Americas, tobacco smoking spread around the world due to the highly addictive nature of tobacco products, which has led to global health, economic, and social impacts ever since (Goodman, 2004). Despite efforts to reduce tobacco consumption in developed countries in recent years, smoking remains the leading preventable cause of disease and death worldwide. The health risks of smoking and other tobacco use are well established, with tobacco consumption being linked to a range of diseases, including lung cancer, cardiovascular disease, chronic obstructive pulmonary disease, and many other illnesses (U.S. Department of Health and Human Services, 2014). The World Health Organization (WHO) estimates that tobacco use is responsible for ~8 million deaths per year, with 80% of these deaths now occurring in low- and middle-income countries (https://www.who.int/news-room/fact-sheets/detail/tobacco). This number is expected to increase to >10 million deaths per year by 2030 if current trends continue.

Nicotine and related pyridine alkaloids, including nornicotine, anatabine, and anabasine, are specialized metabolites present in *Nicotiana* species (Kaminski *et al.*, 2020). In most tobacco varieties, nicotine is the predominant alkaloid, typically comprising >90% of the total alkaloid pool (Sisson and Saunders, 1982). The predominant naturally occurring form of nicotine is an optically pure (*S*)-isomer. Nicotine was first isolated by a German chemist Wilhelm Heinrich Posselt and named in honor of Jean Nicot, a French ambassador who introduced tobacco to Europe in the 16th century (Goodman, 2004). When inhaled, nicotine is rapidly absorbed into the bloodstream through the lungs and can easily pass through the blood–brain barrier to reach the brain. Nicotine is a psychoactive substance that has multiple effects on the brain, acting as an agonist for nicotinic acetylcholine receptors (Benowitz, 2009). One of its main effects is to stimulate the release of dopamine, a neurotransmitter that is involved in the brain’s reward system. This leads to feelings of pleasure, euphoria, and stimulatory motivation, thereby contributing to the strong addictive property of tobacco alkaloids. Over time, regular nicotine use can lead to addiction and tolerance, requiring higher doses to achieve the same effects.

The WHO has recommended reducing nicotine levels in cigarettes to a non-addictive level (0.4 mg g^–1^), which could substantially benefit public health by reducing the number of people who become addicted to tobacco products and by making it easier for smokers to quit the harmful habit (WHO, 2015). In 2022, the US Food and Drug Administration (FDA) proposed a new rule that would establish a maximum nicotine level in cigarettes and other tobacco products sold in the USA in line with the WHO recommendation (https://www.fda.gov/news-events/press-announcements/fda-announces-plans-proposed-rule-reduce-addictiveness-cigarettes-and-other-combusted-tobacco). It is important to develop tobacco varieties with non-addictive nicotine levels to support this public health goal (Lewis, 2019).

Nicotine is produced primarily in the roots of tobacco plants and transported via the xylem to the leaves, where it is stored in vacuoles and serves as a defense mechanism against predation. Due to its potent toxicity, nicotine has historically been used as an insecticide. Insect herbivory and physical damage to the plant can increase nicotine accumulation. The plant hormone jasmonate (JA) plays a crucial signaling role in eliciting nicotine biosynthesis in response to damage, activating transcriptional regulators that coordinate the expression of downstream metabolic and transport genes. The amount of nicotine in tobacco leaves is influenced by various factors such as cultivation practices, environmental conditions, and genetic background. With advances in molecular and genomics research (Dewey and Xie, 2013; Sierro *et al.*, 2014; Wang and Bennetzen, 2015; Shoji, 2020b), it is now possible to use mutational breeding, genetic engineering, and genome editing to generate tobacco plants with low-nicotine or ultra-low nicotine traits, which are defined here as <20% and 5% relative to the level in wild-type tobacco, respectively (Lewis, 2019). Here we overview the recent advances in genetic manipulation of nicotine production in tobacco, presenting an excellent example of plant metabolic engineering with social impacts.

## Alkaloid biosynthesis in tobacco

**Fig. 1.**
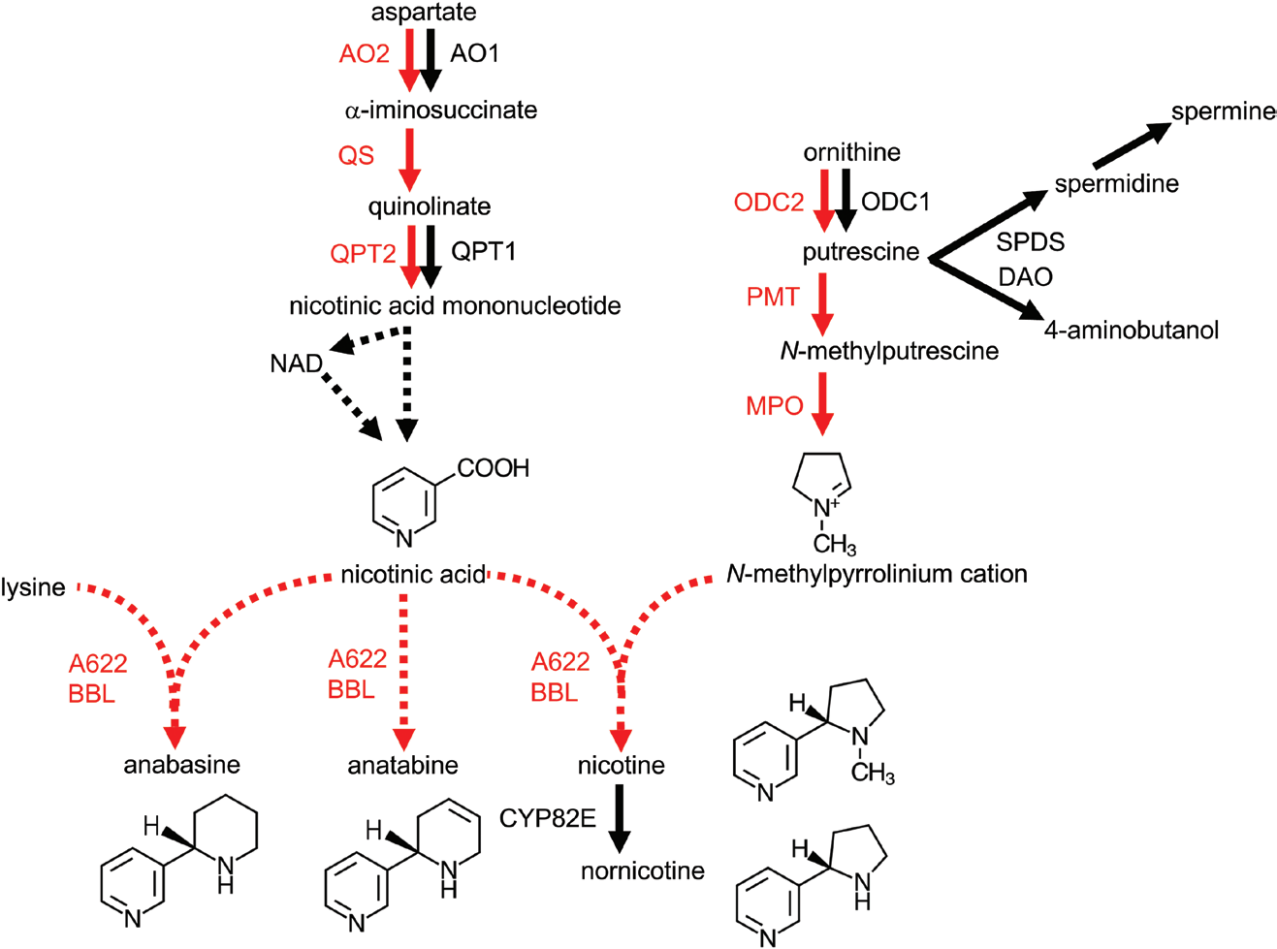
Biosynthesis of nicotine and related alkaloids in tobacco. Dashed arrows represent undefined or multiple steps. Metabolic enzymes whose genes are regulated by ERF199 and ERF189, and their reaction steps, are shown in red. Putrescine *N*-methyltransferase (PMT) and *N*-methylputrescine oxidase (MPO) have been proposed to have evolved from spermidine synthase (SPDS) and diamine oxidase (DAO), respectively. AO, aspartate oxidase; BBL, berberine bridge enzyme-like protein; ODC, ornithine decarboxylase; QPT, quinolinate phosphoribosyltransferase; QS, quinolinate synthase.

Alkaloids are a class of nitrogen-containing, mostly alkaline chemicals that are usually biosynthesized from amino acids and their derivatives through a series of reactions catalyzed by metabolic enzymes from various protein families (Shoji, 2020a). Nicotine is composed of a pyrrolidine and a pyridine ring, which are formed from amino acid precursors early in the pathway and are condensed in later steps (Fig. 1). Most, but not all, of the metabolic genes involved in nicotine biosynthesis have been identified and intensively studied for genetic manipulation (Dewey and Xie, 2013; Shoji, 2020b).

### Pyrrolidine formation

To form pyrrolidine, a five-membered ring intermediate, *N*-methylpyrrolinium cation, is biosynthesized from ornithine through a series of consecutive reactions catalyzed by three enzymes: ornithine decarboxylase (ODC) (Imanishi *et al.*, 1998), putrescine *N*-methyltransferase (PMT) (Hibi *et al.*, 1994), and *N*-methylputrescine oxidase (MPO) (Heim *et al.*, 2007; Katoh *et al.*, 2007) (Fig. 1).

PMT and MPO are suggested to have evolved from primary metabolic enzymes involved in polyamine biosynthesis, specifically spermidine synthase (SPDS) and diamine oxidase (DAO), respectively (Junker *et al.*, 2013; Naconsie *et al.*, 2014). The pyrrolidine-forming branch of nicotine biosynthesis is thought to have arisen through duplication of the polyamine pathway (Kajikawa *et al.*, 2017a, b; Xu *et al.*, 2017). This duplication event resulted in more *ODC* genes, which potentially facilitated enhanced metabolic flow into the duplicated branch. Consequently, this increase in metabolic flow allowed catalytic innovation through the neo-functionalization of the duplicate genes, leading to the evolution of *PMT* and *MPO* genes. There are two *ODC* genes, *ODC1* and *ODC2*, and they exhibit differential expression patterns (Xu *et al.*, 2004). *ODC2* is co-regulated with other genes involved in nicotine biosynthesis, suggesting a coordinated regulation of nicotine production. On the other hand, *ODC1* expression is nearly constitutive, indicating its involvement in basal cellular processes.

Down-regulating both *ODC* genes using RNAi resulted in reduced nicotine levels but increased anatabine accumulation in hairy roots and transgenic plants (DeBoer *et al.*, 2011a; Dalton *et al.*, 2016). Additionally, the *ODC*-silenced lines showed reduced accumulation of polyamines along with various physiological and morphological abnormalities, including early senescence and partial sterility (Dalton *et al.*, 2016; Choubey and Rajam, 2017). These abnormalities are probably caused by the decreased levels of polyamines resulting from *ODC* down-regulation.

PMT catalyzes the first committed step of pyrrolidine formation in nicotine biosynthesis. Several studies have shown that when *PMT* is down-regulated using co-suppression, antisense, or RNAi techniques, there is a significant decrease in nicotine accumulation in *Nicotiana* species (Sato *et al.*, 2001; Chintapakorn and Hamill, 2003; Steppuhn *et al.*, 2004; Wang *et al.*, 2008, 2009). Interestingly, *PMT* knockdown results in a simultaneous increase in anatabine levels (Chintapakorn and Hamill, 2003; Steppuhn *et al.*, 2004; Wang *et al.*, 2009). Additionally, in *PMT*-suppressed lines of *Nicotiana sylvestris*, abnormal leaf fusion and a significant increase in polyamine levels were observed alongside reduced nicotine production (Sato *et al.*, 2001).

Down-regulating *MPO* through RNAi resulted in significant decreases in nicotine and nornicotine levels, accompanied by substantial increases in anatabine and anabasine in tobacco hairy roots (Shoji and Hashimoto, 2008). Anatabine and anabasine do not possess a pyrrolidine ring, indicating that suppressing the expression of *ODC*, *PMT*, or *MPO* genes, involved in pyrrolidine biosynthesis, prompts compensatory production of the pyridine-derived alkaloids.

### Pyridine formation

Nicotinic acid is a primary metabolite in the biosynthesis pathway that supplies NAD, an essential cofactor for many oxidation–reduction reactions. The pyridine part of nicotine and other related alkaloids is derived from nicotinic acid. In the NAD pathway, aspartate is converted to nicotinic acid mononucleotide via quinolinate through reactions catalyzed by aspartate oxidase (AO), quinolinate synthase (QS), and quinolinate phosphoribosyltransferase (QPT) (Sinclair *et al.*, 2000; Katoh *et al.*, 2006) (Fig. 1). Duplication of *AO* and *QPT* genes has occurred in *Nicotiana* species but not in other lineages, enabling massive downstream nicotine production by increasing upstream metabolic flows (Kajikawa *et al.*, 2017a, b; Xu *et al.*, 2017). One of the two *QPT* genes, *QPT2*, is expressed in the roots and induced in response to JA along with other genes involved in nicotine biosynthesis, while *QPT1* is constitutively expressed in all tobacco tissues, possibly involved in NAD biosynthesis (Shoji and Hashimoto, 2011a). Such differential expression patterns between the two genes suggest sub-functionalization after gene duplication. It remains unclear how nicotinic acid is supplied from nicotinic acid mononucleotide.

Down-regulating *AO* genes using RNAi resulted in reduced nicotine accumulation and early senescence in tobacco leaves (Hidalgo Martinez *et al.*, 2020). However, there have been no reports on how manipulating *QS* affects nicotine accumulation or phenotypic traits in tobacco.

QPT is a rate-limiting enzyme in pyridine ring formation. Down-regulating *QPT1* and *QPT2* via RNAi significantly reduced nicotine and anabasine levels and also caused notable changes in plant growth (Khan *et al.*, 2017). These alterations in growth could be attributed to a decreased supply of NAD. Additionally, a *QPT2*-knockout mutant generated using clustered regularly interspaced short palindromic repeats (CRISPR)/CRISPR-associated nuclease 9 (Cas9) genome editing showed drastically reduced nicotine production (Smith *et al.*, 2022). It is important to note that this tobacco mutant displayed only modest growth changes in a greenhouse environment, but, when transplanted to fields, its growth and development were severely inhibited. These findings suggest that knocking out *QPT2* is not a viable strategy for producing agriculturally useful tobacco with low nicotine.

### Condensation of pyrrolidine and pyridine rings

In contrast to the early steps of the nicotine biosynthesis pathway, which largely overlap with or are evolutionarily related to polyamine or NAD pathways, it is unclear how the rings are condensed in the late part of the pathway. It is even unclear whether the substrate for the condensation reaction is nicotinic acid itself or one of its derivatives. Two oxidoreductases, A622 and berberine bridge enzyme-like protein (BBL), have been proposed to be involved in the later steps, but biochemical details of their reactions in the pathway remain to be determined (Hibi *et al.*, 1994; DeBoer *et al.*, 2009; Kajikawa *et al.*, 2009, 2011) (Fig. 1). A622 is an NADPH-dependent reductase belonging to the PIP family (Kajikawa *et al.*, 2009), which was named after its founding members: pinoresinol–lariciresinol reductase (Min *et al.*, 2003), isoflavone reductase (Wang *et al.*, 2006), and phenylcoumaran benzylic ether reductase (Min *et al.*, 2003). BBL is primarily localized in vacuoles within tobacco roots (Kajikawa *et al.*, 2009). A622 and BBL are required to produce all pyridine alkaloids, suggesting that steps mediated by A622 and BBL are shared among the routes leading to the different alkaloids (DeBoer *et al.*, 2009; Kajikawa *et al.*, 2009, 2011) (Fig. 1).

In wood tobacco (*Nicotiana glauca*), silencing of *A622* via RNAi significantly decreased the levels of its predominant alkaloid, anabasine, in the leaves and hairy roots (DeBoer *et al.*, 2009). Furthermore, in tobacco hairy roots, down-regulating *A622* through RNAi drastically reduced the levels of nicotine and other alkaloids, and inhibited root growth (Kajikawa *et al.*, 2009). This growth inhibition was attributed to the overaccumulation of cytotoxic nicotinic acid (Li *et al.*, 2017), not all of which could be utilized for nicotine production.

The tobacco genome contains six *BBL* genes: *BBLa*, *BBLb*, *BBLc*, *BBLd*, *BBLd2*, and *BBLe* (Kajikawa *et al.*, 2017b). Simultaneous RNAi of *BBLa*, *BBLb*, and *BBLc* resulted in a significant reduction of up to 94% in foliar nicotine levels (Kajikawa *et al.*, 2011). Furthermore, careful phenotypic analysis of knockouts of all six *BBL* genes, generated through chemically induced mutagenesis and CRISPR/Cas9-mediated editing (Lewis *et al.*, 2015, 2020; Schachtsiek and Stehle, 2019), revealed that combined mutations in *BBLa*, *BBLb*, and *BBLc* substantially reduced (up to 17-fold) nicotine accumulation (Lewis *et al.*, 2015, 2020). However, additional mutations in *BBLd*, *BBLd2*, and *BBLe* did not contribute to further decreases in nicotine levels (Lewis *et al.*, 2020). Interestingly, tobacco lines with loss of BBL function overaccumulated a minor alkaloid called dehydrometanicotine (Kajikawa *et al.*, 2011; Lewis *et al.*, 2015). Additionally, field-grown *BBL* RNAi lines showed a significant 29% reduction in yield compared with wild-type tobacco, indicating that loss of BBL function negatively affects plant growth (Lewis *et al.*, 2020).

### Nornicotine formation

Another alkaloid of tobacco, nornicotine, typically comprises ~2–5% of the total alkaloids in leaves (Sisson and Saunders, 1982). The conversion of nicotine to nornicotine occurs through a demethylation step catalyzed by nicotine *N*-demethylase, a cytochrome P450 enzyme belonging to the CYP82E subfamily (Fig. 1). Several *CYP82E* genes have been identified in tobacco, including *CYP82E2*, *CYP82E3*, *CYP82E4*, *CYP82E5*, *CYP82E10*, and *CYP82E21*. *CYP82E2* and *CYP82E3* encode non-functional enzymes due to substitutions in critical amino acid residues, while their counterparts in progenitor species *N. sylvestris* and *Nicotiana tomentosiformis* encode highly active demethylases (Siminszky *et al.*, 2005; Gavilano *et al.*, 2007). *CYP82E4* is induced during leaf senescence and curing, and is responsible for the majority of nornicotine production in tobacco (Siminszky *et al.*, 2005; Chakrabarti *et al.*, 2008), whereas *CYP82E5*, *CYP82E10*, and *CYP82E21* are primarily expressed in green leaves, roots, and flower ovaries, respectively, and contribute to nornicotine production to a lesser extent (Gavilano and Siminszky, 2007; Lewis *et al.*, 2010; Liedschulte *et al.*, 2016). The relaxed substrate specificity of the CYP82E4 enzyme is responsible for an increased ratio of (*R*)-nornicotine to (*S*)-nornicotine, which is higher than the ratio of the corresponding nicotine isomers (Cai *et al.*, 2012). In a proportion of individuals in tobacco populations, nornicotine occasionally becomes the dominant alkaloid due to transcriptional reactivation of *CYP82E4*, which is normally silenced (Griffith *et al.*, 1955; Siminszky *et al.*, 2005). However, the genetic or epigenetic basis of this change remains elusive.

Knockout mutations in *CYP82E4*, *CYP82E5*, and *CYP82E10* were obtained from chemically mutagenized populations (Lewis *et al.*, 2010; Song *et al.*, 2020), and the relative contributions of each mutation to nornicotine production were evaluated in a series of mutant genotypes (Lewis *et al.*, 2010). In tobacco, a triple knockout line had significantly decreased nornicotine accumulation in leaves compared with the wild type, which accounted for <1% of the total alkaloid level (Lewis *et al.*, 2010; Song *et al.*, 2020), similar to the results of RNAi-mediated silencing of these three genes in tobacco (Lewis *et al.*, 2008). In a *cyp82e4 cyp82e5 cyp82e10* triple mutant of a flue-cured tobacco variety, along with the decreased nornicotine, nicotine levels were also significantly reduced to 73% of that in the wild type (Song *et al.*, 2020). This reduction was attributed to the down-regulation of nicotine biosynthesis genes, indicating an unknown negative regulatory relationship between nicotine demethylation and nicotine biosynthesis.

Under conditions where the availability of one-carbon (C1) units is limited due to a restricted C1 pool, methyl groups from nicotine become important in C1 metabolism. When a key metabolic gene encoding methylenetetrahydrofolate reductase (MTHFR) involved in tetrahydrofolate-mediated C1 metabolism was down-regulated using RNAi, *CYP82E4* expression was induced possibly to supply methyl groups from nicotine (Hung *et al.*, 2013). Conversely, when *MTHFR* was overexpressed, *CYP82E4* was suppressed, leading to a decrease in nornicotine accumulation (Hung *et al.*, 2013).

### Tobacco-specific nitrosamines

Tobacco-specific nitrosamines (TSNAs) comprise a group of carcinogenic compounds that are formed from the reaction of nitrite and secondary amines, including nicotine and nornicotine, during tobacco curing and use (Konstantinou *et al.*, 2018). TSNAs are highly carcinogenic and mutagenic and have been linked to various types of cancer, particularly lung cancer. The most abundant TSNAs in tobacco are *N*ʹ-nitrosonornicotine (NNN), 4-(methylnitrosamino)-1-(3-pyridyl)-1-butanone (NNK), and *N*-nitrosoanatabine (NAT). NNN, which is readily formed from nornicotine, is considered one of the most carcinogenic compounds in tobacco products and more harmful than other TSNAs. Suppressing nornicotine formation is an effective strategy to restrict NNN generation (see ‘Nornicotine formation’).

Nitrate is a nitrosating agent that contributes to TSNA formation. Therefore, in addition to reducing precursor alkaloids, lowering nitrate levels in tobacco leaves is a promising strategy to reduce the formation of these carcinogenic compounds. Burley tobacco varieties accumulate high levels of nitrate (Lewis *et al.*, 2012), making them a prime target for this strategy. Leaf nitrate and TNSA levels were effectively reduced by expressing a constitutively active variant of nitrate reductase, a key enzyme in the nitrogen assimilation pathway (Lu *et al.*, 2016). Additionally, a member of the chloride channel (CLC) protein family mediates nitrate accumulation in the vacuole (Geelen *et al.*, 2000). Suppressing or knocking out a tobacco *CLC* gene *CLCNt2* resulted in decreased nitrate and TSNA levels in cured leaves without negatively impacting biomass (Bovet *et al.*, 2022).

## Membrane transporters

Membrane transporters from various families play crucial roles in facilitating the movement of natural products across biological membranes. In tobacco cells, nicotine is sequestered into vacuoles to prevent cytotoxic effects at high concentrations. Several transporters belonging to the multidrug and toxic compound extrusion (MATE) family, namely JASMONTE-INDUCIBLE ALKALOID TRANSPORTER 1 (JAT1), JAT2, MATE1, and MATE2, mediate vacuolar sequestration of nicotine (Morita *et al.*, 2009; Shoji *et al.*, 2009; Shitan *et al.*, 2014). These MATE transporters are localized in the tonoplast membrane and function as proton antiporters, coupling proton gradients across the membrane with nicotine uptake into the vacuoles. *JAT1* and *JAT2* are expressed in the leaves and encode proteins that are phylogenetically related to the xenobiotic-transporting DETOXIFICATION1 (DTX1) transporter found in Arabidopsis (*Arabidopsis thaliana*) (Morita *et al.*, 2009; Shitan *et al.*, 2014). *MATE1* and *MATE2* encode homologs of flavonoid transporters and are co-expressed with nicotine biosynthesis genes in tobacco roots (Shoji *et al.*, 2009). *MATE2* is located near *A622* on chromosome 12 in the tobacco genome (Kajikawa *et al.*, 2017b). It is worth noting that this clustering of non-homologous genes is a unique case so far in the nicotine pathway. The crystal structure of MATE2 has been reported, providing valuable insights into its substrate recognition and transport mechanisms (Tanaka *et al.*, 2021). However, it should be noted that RNAi-mediated suppression of *MATE1* and *MATE2* did not significantly alter alkaloid levels in the leaves and roots (Shoji *et al*., 2009). As for *JAT1* and *JAT2*, it remains unknown whether manipulating these transporter genes impacts alkaloid profiles.

NICOTINE UPTAKE PERMEASE 1 (NUP1) is a member of the purine permease family localized in the plasma membrane (Hildreth *et al.*, 2011; Kato *et al.*, 2014) and functions as a transporter responsible for the uptake of metabolites containing a pyridine ring, including nicotine and vitamin B6 (Hildreth *et al.*, 2011; Kato *et al.*, 2014, 2015). *NUP1* is primarily expressed in epidermal cells of roots (Kato *et al.*, 2014). It has been suggested that NUP1 may modulate nicotine biosynthesis by participating in the transcriptional regulation of the ethylene response factor (ERF) transcription factor genes *ERF199* and *ERF189*, as well as in root growth (Hildreth *et al.*, 2011; Kato *et al.*, 2014). However, the exact mechanisms linking membrane transport and these regulatory functions are still not understood.

A grafting experiment demonstrated root-to-shoot translocation of tobacco alkaloids between tobacco rootstock and tomato scion (Dawson, 1942). Nicotine moves upward through the xylem along the transpiration stream. Nicotine efflux occurs from root cells, and nicotine influx takes place in leaf cells, facilitating xylem loading and unloading, respectively. However, the membrane transporters responsible for these processes have remained elusive. Some wild *Nicotiana* species lack or have reduced abilities for long-distance alkaloid translocation (Pakdeechanuan *et al.*, 2012). Exploring the genetic basis of such natural variations is intriguing and could provide valuable insights into the alkaloid transport mechanism.

## Regulation

In plants, metabolic pathways that produce specialized products are subject to dynamic regulation in response to developmental and environmental cues. Transcription factors often coordinate the expression of multiple metabolic and transport genes involved in these pathways and play key roles in integrating the metabolic processes in various biological responses (Chezem and Clay, 2016; Shoji *et al.*, 2021).

**Fig. 2.**
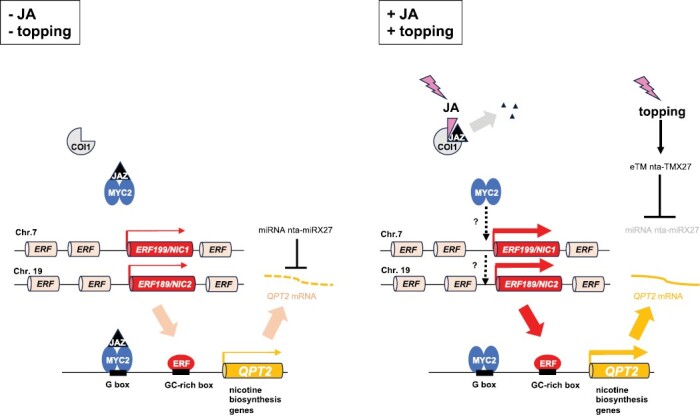
A model of JA- and topping-dependent induction of nicotine biosynthesis genes in tobacco. ERF199 and ERF189 up-regulate nicotine biosynthesis genes (e.g. *QPT2*) by recognizing GC-rich elements in their promoter regions. A bHLH-family MYC2 transcription factor mediates JA-dependent induction of *ERF* genes. MYC2 is a direct target of JAZ repressors. When a co-complex comprising COI1 and JAZ perceives a JA signal, JAZ proteins are removed through proteasome-dependent degradation. MYC2 also directly up-regulates nicotine biosynthesis genes by recognizing G-box elements as homo- and/or heterodimers. Topping induces nta-TMX27 expression, leading to nta-miRX27 degradation. Degradation of this miRNA relieves its inhibition of *QPT2*, resulting in enhanced *QPT2* expression.

In tobacco, the JA signaling pathway up-regulates defense-related nicotine biosynthesis (Shoji *et al.*, 2000). JAs comprise a group of plant hormones derived from fatty acids that play a central role in development and defense responses against biotic and abiotic stresses (Wasternack and Strnad, 2018). JAs have been widely used as elicitors to induce the production of natural products in plant tissue cultures. The molecular mechanism of the JA signaling pathway, from signal perception to gene induction, is conserved in a wide range of plant species (Paschold *et al.*, 2007; Shoji *et al.*, 2008). The JA signaling pathway involves the proteasome-dependent degradation of JASMONATE ZIM-DOMAIN (JAZ) repressors, which leads to the activation of transcription factors such as the basic helix–loop–helix (bHLH)-family MYC2 (Wasternack and Strnad, 2018) (Fig. 2). However, it remains unclear how the conserved upstream JA signaling cascade is linked to diverse downstream defense responses and metabolic pathways, such as the nicotine biosynthesis pathway in tobacco. The JA-responsive transcription factors ERF199 and ERF189 have emerged as a central molecular component linking upstream signaling with downstream metabolic processes in tobacco (Shoji and Yuan, 2021) (Fig. 2).

### ERF199 and ERF189 transcription factors

In the 1930s, a mutant of a Cuban cigar tobacco variety with low nicotine content was discovered in Europe. This naturally occurring mutant has since been used to breed commercial tobacco varieties with low-nicotine traits (Legg *et al.*, 1970), which typically have nicotine levels that are 10–20% of those found in wild-type tobacco. The low-nicotine phenotype is determined by semi-dominant mutations at two unlinked genetic loci, *NICOTINE1* (*NIC1*) and *NICOTINE2* (*NIC2*), which are also referred to as *A* and *B*, respectively (Legg and Collins, 1971). However, it should be noted that the low-nicotine tobacco resulting from these mutations has certain drawbacks, such as inferior leaf quality and increased susceptibility to insect pathogens (Chaplin and Weeks, 1976). As a result, the economic value and utilization of this genotype have been limited.

Molecular genetics and genomics studies have revealed the identities of the *NIC* genes. *NIC1* was identified as *ERF199*, located on chromosome 7 and derived from *N. sylvestris* (Qin *et al.*, 2021; Shoji *et al.*, 2022), while *NIC2* was identified as *ERF189*, located on chromosome 19 and derived from *N. tomentosiformis* (Shoji *et al.*, 2010; Kajikawa *et al.*, 2017b) (Fig. 2). *ERF199* and *ERF189* encode JA-responsive ERF transcription factors and are mostly coordinately expressed with nicotine biosynthesis genes in response to various developmental and phytohormonal cues. In the tobacco genome, *ERF199* and *ERF189* are present in clusters of five and ten homologous *ERF* genes, respectively (Kajikawa *et al.*, 2017b) (Box 1). Based on expression and functional studies, it is reasonable to consider that ERF199 and ERF189 mainly regulate nicotine biosynthesis (Shoji and Hashimoto, 2015). ERF199 and ERF189 up-regulate numerous nicotine biosynthesis genes, including *ODC2*, *PMT*, *MPO*, *AO2*, *QS*, *QPT2*, *A622*, *BBL*, *MATE1*, and *MATE2*, by recognizing specific GC-rich *cis*-regulatory elements in the promoter regions of the downstream target genes (Shoji *et al.*, 2010, 2013). *ERF189*, and possibly *ERF199*, are up-regulated by the MYC2 transcription factor, thereby linking upstream JA signaling to downstream nicotine biosynthesis (Shoji and Hashimoto, 2011b; Sui *et al.*, 2021) (Fig. 2).

Box 1. **Conserved transcriptional regulators involved in metabolism of defense compounds in multiple plant species**

A small group of ERF transcription factors, classified in clade II of group IXa, including ERF199 and ERF189 involved in nicotine biosynthesis in tobacco, have emerged as key transcriptional regulators of JA-induced metabolism of defense compounds in various plant lineages. These ERFs include ORCAs, which regulate biosynthesis of monoterpenoid indole alkaloids in periwinkle (*Catharanthus roseus*) (van der Fits and Memelink, 2000), JRE4, which regulates steroidal glycoalkaloid production in tomato (*Solanum lycopersicum*) (Cardenas *et al.*, 2016; Thagun *et al.*, 2016; Nakayasu *et al.*, 2018), and ORA, which regulates artemisinin production in sweet annie (*Artemisia annua*) (Lu *et al.*, 2013). Most of these ERF genes form clusters of homologs in plant genomes. As more plant genomes become available, clustered ERF genes of this group have been discovered in a wide range of eudicots (Shoji and Yuan, 2021). It remains an open question whether these newly discovered ERFs also function as regulators of defense compound metabolism or other processes. Implications of these findings on the evolution of metabolic pathways were discussed elsewhere (Shoji, 2019).

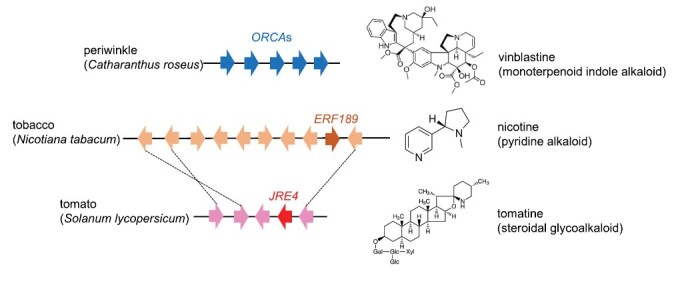

Clustered ERF transcription factor genes and their target specialized metabolites. Clusters of *ERF* genes in three plant species are depicted, with closely related genes in tobacco and tomato linked by broken lines. Specialized metabolites regulated by these *ERF* genes are shown on the right.

Disrupting *ERF199* and *ERF189* simultaneously using CRISPR/Cas9 gene editing resulted in an ultra-low-nicotine phenotype in tobacco, with nicotine levels reaching only 2–5% of the wild-type levels. Importantly, this was achieved without causing major growth defects, at least in a greenhouse environment (Hayashi *et al*., 2020). A comprehensive metabolic profiling revealed significant influences of this double knockout not only on nicotine but also on other metabolites that are not directly related to the nicotine biosynthesis pathway in tobacco. This implies the existence of unknown regulatory mechanisms within the metabolic network (Hayashi *et al*., 2020). To determine the potential utility of the double knockout plants in agricultural fields, it will be necessary to fully characterize their agronomic properties, including comparing them with the naturally occurring *nic1 nic2* double mutant in terms of leaf quality, insect resistance, and other agronomic traits (Chaplin and Weeks, 1976). Moreover, complete loss of function of ERF199 resulted in a low-nicotine phenotype similar to that observed in the *nic1 nic2* double mutant, emphasizing the importance of ERF199 as a regulator of nicotine biosynthesis (Burner *et al.*, 2022; Shoji *et al.*, 2022). In contrast, a null mutation of *ERF189* only slightly affected nicotine levels (Shoji *et al*., 2022).

*ERF199* and *ERF189* are highly expressed in the roots, where nicotine is produced, but have nominal expression levels in the leaves (Hayashi *et al*., 2020; Kajikawa *et al*., 2017a, b). Interestingly, transient overexpression of *ERF189* in *Nicotiana benthamiana* leaves led to the hyperaccumulation of nicotine, suggesting that ERF189, or possibly ERF199, is not only necessary but also sufficient for inducing alkaloid production in the leaves of *Nicotiana* plants (Hayashi *et al.*, 2020). Further characterization is required to elucidate the molecular basis of functional redundancy and potential differentiation between these two ERFs.

ERF32, ERF91, and ERF221, which are phylogenetically related to ERF199 and ERF189, are also reported to have regulatory roles in nicotine biosynthesis, primarily based on gain-of-function and promoter binding analyses (DeBoer *et al.*, 2011b; Sears *et al.*, 2014; Liu *et al.*, 2019; Sui *et al.*, 2019). However, additional genetic evidence obtained through knockout and other experiments is required to confirm their *in planta* roles in regulating nicotine biosynthesis.

### MYC2 transcription factor

The MYC2 transcription factor plays a central role in the JA signaling cascade, regulating a wide range of genes involved in JA-dependent responses (Luo *et al.*, 2023). This bHLH-family transcription factor is encoded by four genes in the tobacco genome: *MYC1a*, *MYC1b*, *MYC2a*, and *MYC2b* (Shoji and Hashimoto, 2011b; Zhang *et al.*, 2012). In addition to regulating other target genes, the tobacco MYC2 transcription factors up-regulate numerous nicotine biosynthesis genes directly by recognizing G-box elements in their promoters and indirectly through ERF199 and ERF189 (DeBoer *et al.*, 2011b; Shoji and Hashimoto, 2011b; Zhang *et al.*, 2012) (Fig. 2).

Disrupting *MYC2a* in tobacco resulted in an 80% reduction in foliar nicotine levels (Sui *et al.*, 2021). This finding highlights the importance of MYC2a in regulating nicotine biosynthesis and suggests that there may be functional differences among the various MYC2 homologs. Alternatively, it is possible that MYC2 members in tobacco form heterodimers, similar to their homologs in other species (Fernandez-Calvo *et al.*, 2011). In this case, knocking out any one *MYC2* member could drastically affect nicotine levels, acting in a dominant-negative manner. It will be important to carefully examine the impacts of the loss of each *MYC2* gene individually to determine their specific contributions to nicotine biosynthesis and overall plant physiology, along with their potential interactions.

Apart from the ERFs and MYC2 mentioned above, several other transcription factors belonging to the ARF, MYB, NAC, and WRKY families have also been implicated in regulating nicotine biosynthesis in various contexts (Fu *et al.*, 2013; Jin *et al.*, 2017; Hu *et al.*, 2021; Bian *et al.*, 2022). To understand the *in planta* regulatory roles of these transcription factors and their relationship with ERFs and MYC2 in JA-mediated regulation of the nicotine pathway, further genetic and other analyses are necessary.

### Protein phosphorylation

Transcription factors are often regulated through protein phosphorylation. A mitogen-activated protein (MAP) kinase kinase JAM1, a MAP kinase MPK4, and a protein phosphatase PP2C2b have been proposed to mediate the regulation of nicotine biosynthesis in tobacco (DeBoer *et al.*, 2011b; Liu *et al.*, 2021). Further analysis is necessary to identify the specific targets of phosphorylation and their functional relationship with ERFs and the JA signaling cascade. Understanding the upstream regulatory components and their interactions is crucial for effectively manipulating the nicotine biosynthesis pathway.

### Regulatory non-coding RNAs

miRNAs and long non-coding RNAs (lncRNAs) are important regulatory factors in various biological processes, including in regulating specialized metabolism in plants (Owusu Adjei *et al.*, 2021; Wang *et al.*, 2023; Zhan and Meyers, 2023). miRNAs are small non-coding RNAs, typically 20–24 nucleotides long, that can induce degradation of target mRNAs or repress their translation (Zhan and Meyers, 2023). lncRNAs exhibit diverse mechanisms of gene regulation, many of which are still not fully understood (Wang *et al.*, 2023). One type of lncRNA, called endogenous target mimics (eTMs), act as decoys by mimicking the target RNAs of specific miRNAs, inhibiting the actions of the miRNAs (Wu *et al.*, 2013).

In tobacco, comprehensive screenings of non-coding RNA species have revealed the relevance of some of these RNAs in regulating genes in the nicotine pathway (Chen *et al.*, 2019; Jin *et al.*, 2020; Zheng *et al.*, 2022). One pair of regulatory RNAs, the miRNA nta-miRX27 and its corresponding eTM nta-TMX27, target a key nicotine biosynthesis gene, *QPT2*, to regulate nicotine levels in tobacco (Li *et al.*, 2015). Removing the uppermost growing point of the plant (called ‘topping’) is a common practice in tobacco cultivation that stimulates nicotine production (Qin *et al.*, 2020). Following topping, nta-TMX27 expression is induced, which leads to nta-miRX27 degradation (Li *et al.*, 2015) (Fig. 2). Degradation of the miRNA relieves its inhibition on *QPT2*, resulting in enhanced *QPT2* expression (Li *et al.*, 2015). Altering the expression of nta-miRX27 and nta-TMX27 significantly affects nicotine accumulation (Li *et al.*, 2015).

### Environmental and hormonal regulations

Several studies have reported the influence of environmental stresses, including high temperature (Yang *et al.*, 2016), flooding (Zhang *et al*., 2016), and salt stress (Chen *et al.*, 2016b), on nicotine accumulation in tobacco. High temperature induces the expression of nicotine biosynthesis genes through the JA signaling pathway (Yang *et al.*, 2016). Additionally, carbon monoxide (Cheng *et al.*, 2018) and hydrogen sulfide (Chen *et al.*, 2016a) have been suggested to act as signals in this induction process. However, the physiological and ecological significance of these stress-induced responses in nicotine accumulation remain unclear.

Ethylene treatment in *Nicotiana attenuata* and *N. sylvestris* roots clearly suppressed the JA-dependent induction of nicotine biosynthesis genes (Shoji *et al*., 2000; Winz and Baldwin, 2001). Additionally, ethylene signaling inhibits the JA-mediated induction of *ERF189* in tobacco (Shoji *et al*., 2010). The nicotine accumulation stimulated by topping is believed to be caused by reduced auxin supply from apical tissues. Indeed, auxin application leads to rapid down-regulation of *PMT* and *A622* expression in tobacco roots, which reduces nicotine biosynthesis (Hibi *et al*., 1994).

## Conclusions and perspectives

Recent molecular and genomic studies have made substantial contributions to our understanding of nicotine biosynthesis and its regulation in tobacco. These studies have revealed numerous metabolic, transport, and regulatory genes that play key roles in nicotine accumulation. The discovery of these genes provides valuable genetic tools for manipulating and controlling alkaloid production and accumulation in tobacco plants.

All metabolic genes involved in the formation of pyridine and pyrrolidine rings have been identified. However, efforts to reduce alkaloid contents by manipulating these early steps in the nicotine biosynthesis pathway have not always yielded favorable outcomes. There are two main reasons for these failures: (i) interference with polyamine and NAD supplies (Sato *et al.*, 2001; Dalton *et al.*, 2016; Choubey and Rajam, 2017; Khan *et al.*, 2017; Hidalgo Martinez *et al.*, 2020; Smith *et al.*, 2022) and (ii) the compensatory production of pyridine-derived alkaloids, anabasine and anatabine, when the supply of pyrrolidine rings is blocked (Chintapakorn and Hamill, 2003; Steppuhn *et al.*, 2004; Shoji and Hashimoto, 2008; Wang *et al.*, 2009; DeBoer *et al.*, 2011a; Dalton *et al.*, 2016). In contrast to the early steps, biochemical details of later steps in nicotine biosynthesis, including ring condensation, remain elusive. Nevertheless, a breakthrough was achieved via a triple knockout mutant involving three *BBL* genes that participate toward the end of the pathway (Lewis *et al.*, 2015, 2020). This mutant exhibited a drastic reduction in alkaloid content with a moderate decrease in yield.

Nornicotine, a precursor of a carcinogenic TSNA (Konstantinou *et al.*, 2018), is a target for alkaloid reduction in tobacco breeding programs. Nornicotine is formed from nicotine through demethylation catalyzed by CYP82Es. Disrupting three *CYP82E* genes significantly reduced the nornicotine content in tobacco (Lewis *et al.*, 2010; Song *et al.*, 2020). This finding presents a promising strategy for reducing the levels of this harmful compound in tobacco plants.

A homologous pair of JA-responsive ERF transcription factors, ERF199 and ERF189, work together to coordinate the expression of many nicotine metabolic and transport genes in tobacco. Double knockout of *ERF199* and *ERF189* resulted in an ultra-low-nicotine phenotype in tobacco plants without substantial growth defects under greenhouse conditions (Hayashi *et al.*, 2020). However, it will be crucial to evaluate the performance of the double mutant in field settings to determine its suitability for practical applications.

Epigenetic mechanisms include DNA methylation, where methyl groups are added to specific regions of the DNA, and histone modification, where chemical groups are added to histone proteins to alter the chromatin structure. These modifications are heritable through cell divisions but do not involve alterations in the underlying DNA sequences, resulting in gene activation and repression. Regulatory RNA molecules, such as small and long non-coding RNAs, play a significant role in epigenetic regulation. Epigenetics and regulatory RNAs have emerged as crucial mechanisms in the regulation of specialized metabolism in plants (Hayashi *et al.*, 2023). Although largely unexplored, it is intriguing to explore whether and, if so, how epigenetic control is significant in nicotine biosynthesis.

It is important to explore natural variations in alkaloid profiles within and between different *Nicotiana* species. Through such studies, we can gain valuable insights into the genetic and biochemical factors that influence the production and regulation of alkaloids in *Nicotiana* plants. This has been exemplified by the molecular identification of *NIC1* and *NIC2*. A large collection of *Nicotiana* germplasms serves as a valuable genetic resource to investigate the natural diversity of alkaloid profiles (Sisson and Saunders, 1982; Kaminski *et al.*, 2020). This collection encompasses a wide range of *Nicotiana* species and varieties, each with its own unique alkaloid profile (Sisson and Saunders, 1982; Kaminski *et al.*, 2020). By employing powerful genomics approaches, such as high-throughput sequencing (Sierro *et al.*, 2014, 2018; Xu *et al.*, 2017), we can delve deeper into the genetic and epigenetic basis of alkaloid diversity and identify more key genes and regulatory elements involved in alkaloid biosynthesis and regulation.

*Nicotiana* plants have gained attention as a promising platform for plant-based bioproduction due to their robust growth, high biomass accumulation, established cultivation and processing infrastructure, genetic modification potential, and metabolic versatility (Jassbi *et al.*, 2017; Molina-Hidalgo *et al.*, 2021). One important objective in this context is to reduce the production of addictive alkaloids, aiming to decrease the presence of these harmful chemicals in the final products and the surrounding environment. Furthermore, our comprehensive understanding of the genetic framework underlying nicotine production and accumulation provides a valuable foundation for further genetic modification and metabolic engineering of these plants. By integrating synthetic biology and other methodologies, it becomes possible to manipulate the biosynthetic pathways and regulatory networks, paving the way for developing *Nicotiana* plants with enhanced production of the desired compounds.
